# Progress in Gynæcology

**Published:** 1903-09-05

**Authors:** 


### Progress in Gynæcology.

**Primary Cancer of the Fallopian Tube.**—Primary cancer of the Fallopian tube is such a comparatively rare disease that every case is worthy of careful study. Graefe[1] records a case which occurred in a sterile woman aged 51; she had been married 22 years, and apparently had hitherto been free from any pelvic inflammatory disease. The periods, regular till nearly one year before observation, became very profuse, and seemed to reappear after an interval of a week. On vaginal examination a tense sausage-shaped tumour was felt in Douglas' pouch. The patient declined any operation. Two and a half years later she returned to Graefe, when he found that the tumour in Douglas' pouch had not altered in size, but he was able to detect a rounded tumour the size of the fœtal head to the right of the one in Douglas' pouch. He opened the abdomen and removed a cyst of the right ovary which had burrowed deeply into the right broad ligament, and a distended right tube shaped like a tobacco pipe. On opening this he found a papillary tumour the size of a chestnut growing from the inner wall of the ampullary portion of the tube. This proved to be a malignant growth; the epithelium of the villous masses was arranged in several layers, and some of it had invaded the deeper tissues in the tubal wall. The patient was free from recurrence eight months after the operation. The history clearly showed that a papilloma of the tube, which must have been present for some time, had become malignant. Bland-Sutton[2] also records a case in which the disease arose in the tubal ampulla near its ostium, and there was an unusually long unimplicated stretch of tube between it and the uterine cavity. In this case irregular uterine hæmorrhage was a leading symptom, the diseased tube being the source of the blood.

**Melanotic Dermoid Sarcoma of the Ovary.**—Amann[3] reports an interesting case of melanotic dermoid sarcoma of the ovary. The patient was 59 years old, and her hair was remarkable for its intensely black colour. She had been four times pregnant. The tumour had been noticed for six months, it had grown rapidly, and there had been marked loss of flesh. At the operation no adhesions and no extension of the disease beyond the limits of the tumour were detected. The growth was as big as a man's head, and was bluish-black in colour; it had developed around a true ovarian dermoid, which contained fat and a few tufts of hair. The tumour itself showed a dark soft surface on section, which became somewhat convex; it was found to be a true melanotic sarcoma.

**Prolapsus Uteri.**—There is still considerable difference of opinion as to the operative treatment of prolapse of the uterus. E. Stanmore Bishop[4] points out that the normal uterus is one which is free to move in every direction within certain limits but not beyond, and he describes an operation upon the utero-sacral ligaments, by means of which the uterus without being absolutely fixed, is kept in its normal position. The technique of the operation is as follows:—The protrusion being reduced, the patient is placed in the extreme Trendelenburg position, the abdomen opened by a median incision, and the fundus of the uterus held well forward. A special sound is passed up the vagina by an assistant, and made to press upwards the posterior fornix so as to render it prominent. On either side a stout silk thread is passed vertically through the substance of the fornix, avoiding the mucous lining, so that each protruding end is half an inch distant from the other, and the whole loop is from one-half to three-quarters of an inch from the cervix. The fornix is now applied to the sacrum, and a spot is chosen directly opposite, free from vessels, subjacent nerves, and ureter, and well outside the rectum, when the needle carrying this suture is entered deeply so as to include the periosteum covering the bone; it is brought out again half an inch directly above its point of entrance. Before tying this suture, a narrow strip of peritoneum is removed from that portion of the fornix which lies in its grip, so as to bare the connective tissue beneath. This is repeated on the opposite side; the sutures are tied and the ends cut short. Ten cases are recorded in which this operation was performed with very satisfactory results.

**Ovarian Pregnancy.**—It is only during the last few years that the possibility of a primary ovarian pregnancy has been admitted; it being previously considered that all extra-uterine pregnancies were primarily tubal. A case is reported by J. F. Thompson[5] in which the evidence in favour of a primary ovarian pregnancy is conclusive. The patient, age 32, had suffered from irregular attacks of pelvic pain for seven years. Menstruation was regular, but since the last period there had been continuous pain in the lower part of the abdomen, and on examination a tender swelling was found to the left of the uterus. The abdomen was opened and the left ovary found to be enlarged. On its upper and inner border there was a dark red tumour the size of a horse-chesnut. The right ovary was normal. A section through the tumour showed a thick wall made up of blood clot. The method of attachment of the chorion was well seen. A fœtus of about 25 to 30 days old was attached to the wall of the ovisac by a short umbilical cord. A microscopic examination of the tumour proved it to have undoubtedly been a pregnancy. The tube and its fimbriæ were quite distinct and unattached to the tumour. Mendes de Leon and Holleman[6] also report a case. The patient who was a multipara, had to the left side and above the posterior vaginal vault a tumour the size of a fist, connected with the uterus by a long pedicle. Laparotomy disclosed a dark red oval hard tumour, on which was seen an unaltered mass of ovarian substance about 1·5 c m. broad. The remnant of the ovary was marked off from the hæmatoma by an indented line; it did not contain any corpus luteum. Microscopic examination proved the hæmatoma to be the result of an interrupted ovarian pregnancy in a Graafian follicle; it contained chorionic villi in various stages of involution.

**Deciduoma Malignum.**—McCann[7] reports an interesting case of deciduoma malignum in a woman age 53. Menstruation had ceased for 12 months. She then had irregular uterine hæmorrhage for six months, occurring about every four or five days, and being so profuse that she was confined to bed while it lasted. On examination the uterus was found enlarged to the size of a three-months' pregnancy, uniform and soft in consistence and freely movable. The passage of a sound caused most profuse hæmorrhage. The uterus was removed by vaginal hysterectomy, but the patient died on the sixth day from suppression of urine. The entire uterine cavity was filled with recent and old-standing blood clot. Sections cut at different levels showed new growth between the clot and the uterine wall. The microscopic examination confirmed the diagnosis of deciduoma malignum.

**Complete Suppression of Urine in a case of Ovarian Tumour and Pregnancy.**—Suppression of urine from pressure by a non-malignant tumour is very rare, and is due in most cases to pressure on the ureters from pelvic tumours. In this case recorded by Bernard Pitts[8] the pressure appears to have been upon the kidneys and their vessels, and was caused by the combined effects of a very large multilocular ovarian cyst, and a pregnancy of about six months. The patient who had had an abdominal tumour for three years became pregnant for the ninth time. The pregnancy was uneventful until about the third month, when she noticed that she was increasing in size very rapidly, so that by the middle of the fifth she was unable to get about the house. The urine about this time became scanty and albuminous, and 14 days later there was complete suppression for 16 hours. As symptoms of uræmia had set in, it was decided to open the abdomen at once. It was found that the pregnant uterus reached to just above the umbilicus, and was capped and surrounded by a large multilocular ovarian tumour. Owing to the collapsed condition of the patient it was imperative to complete the operation as quickly as possible, and as there was no proper pedicle to the tumour, it was decided to remove the uterus as well. The uterus was therefore divided above the cervix, and the stump of the cervix with the attachment of the tumour stitched to the lower end of the abdominal incision and treated extra-peritoneally. Recovery, although somewhat protracted, was complete. After reviewing the various causes of anuria, Bernard Pitts considers that the most probable cause in this case was pressure on the kidneys and their vessels by the pregnant uterus and tumour.

[1] Brit. Med. Jour., Jan. 10, 1903. [2] Lancet, Nov. 15, 1902. [3] Brit. Med. Jour., Feb. 14, 1903. [4] Lancet, March 14, 1903. [5] Quart. Med. Jour., February 1903. [6] Brit. Gynæco. Jour., February 1903. [7] Jour. of Obstet. and Gynæco. of British Empire, March 1903. [8] Lancet, Jan. 21 1903.

